# The Influence of Climate Change on the Distribution of *Hibiscus mutabilis* in China: MaxEnt Model-Based Prediction

**DOI:** 10.3390/plants13131744

**Published:** 2024-06-24

**Authors:** Lu Zhang, Beibei Jiang, Yu Meng, Yin Jia, Qian Xu, Yuanzhi Pan

**Affiliations:** 1 College of Landscape Architecture, Sichuan Agricultural University, Chengdu 611130, China; sicauzl@outlook.com (L.Z.);; 2 College of Landscape Architecture and Tourism, Hebei Agricultural University, Baoding 071000, China; 3 College of Forestry, Sichuan Agricultural University, Chengdu 611130, China

**Keywords:** climate change, *Hibiscus mutabilis*, MaxEnt model, potential suitable habitats

## Abstract

Our study utilized 374 geographical distribution records of *H. mutabilis* and 19 bioclimatic factors, employing the MaxEnt model and the Geographic Information System (ArcGIS). The key environmental variables influencing the suitable distribution areas of *H. mutabilis* were analyzed through the comprehensive contribution rate, permutation importance, and Pearson correlation coefficient. Based on this analysis, the contemporary and future suitable distribution areas and their extents were predicted. The results indicate that the key limiting factor affecting the suitable distribution areas of *H. mutabilis* is the precipitation of the driest month (bio14), with secondary factors being annual precipitation (bio12), annual mean temperature (bio1), and annual temperature range (bio7). Under contemporary climate conditions, the total suitable area for *H. mutabilis* is approximately 2,076,600 km^2^, primarily concentrated in the tropical and subtropical regions of southeastern China. Under low-to-medium-emission scenarios (SSP1-2.6, SSP2-4.5), the total suitable area of *H. mutabilis* shows a trend of first decreasing and then increasing compared to the current scenario. In contrast, under high-emission scenarios (SSP5-8.5), it exhibits a trend of first increasing and then decreasing. The spatial pattern changes indicate that the retention rate of suitable areas for *H. mutabilis* ranges from 95.28% to 99.28%, with the distribution centers primarily located in Hunan and Guizhou provinces, showing an overall migration trend towards the west and north. These findings suggest that *H. mutabilis* possesses a certain level of adaptability to climate change. However, it is crucial to consider regional drought and sudden drought events in practical cultivation and introduction processes. The results of our study provide a scientific basis for the rational cultivation management, conservation, and utilization of germplasm resources of *H. mutabilis.*

## 1. Introduction

Climate, as a primary controlling factor in ecosystems, has profound impacts on species’ geographical distributions and ecological processes [1,2]. Numerous reports indicate that climate change can lead to restricted growth or death of certain plants [3], further causing habitat fragmentation [4] and even species extinction [5]. Against the backdrop of global climate change, research on species’ suitable habitats has become increasingly urgent. Establishing species distribution models to simulate and predict potential geographical distributions of species has become a crucial method for understanding species–environment relationships, conserving biodiversity, and rationally utilizing species resources.

The application of species distribution models (SDMs) has become a pivotal tool in the study of ecology and conservation biology [6,7]. Among these models, the MaxEnt model, developed by Phillips, is a spatial distribution model at the geographic scale based on the maximum entropy theory [8]. It is highly favored for its efficiency and accuracy, as it integrates limited species presence data with environmental information to effectively understand species distribution patterns and explore their relationships with environmental variables. The MaxEnt model is now widely used to study the impact of climate change on species distribution [9,10], such as *Osmanthus fragrans* [11], *Cotoneaster multi florus* [12], and *Primula filchnerae* [13]. The findings from these studies provide valuable guidance for the conservation of genetic diversity and the management of germplasm resources [14,15], and have further heightened public concern about climate change [16].

*Hibiscus mutabilis* (*H. mutabilis)*, belonging to the genus *Hibiscus* in the family Malvaceae, is native to China and is now widely distributed in tropical and subtropical regions of Asia, Africa, North America, and South America [17]. As a common ornamental plant, it is extensively planted and utilized for its outstanding landscape aesthetics and ecological functions [18,19]. Additionally, the leaves of *H. mutabilis* possess significant medicinal value, typically offering anti-inflammatory, detoxifying, expectorant, antipyretic, and anesthetic effects [20]. Current research on *H. mutabilis*, both in China and internationally, primarily focuses on its medicinal value [21,22], stress tolerance [23,24], and pest and disease management [25,26]. However, there have been no systematic studies on its potential suitable habitats, influencing factors, or possible responses to future climate change.

Our study focuses on *H. mutabilis*, utilizing the MaxEnt model and Geographic Information System (ArcGIS) technology to simulate and predict its suitable habitats in both contemporary and future periods. By investigating the primary bioclimatic factors influencing its geographical distribution and analyzing the spatial variation patterns of its suitable habitats, this research aims to provide theoretical support for the rational cultivation and management of *H. mutabilis*. Additionally, it seeks to offer scientific evidence for the conservation and utilization of its germplasm resources.

## 2. Materials and Methods

### 2.1. Data Collection

#### 2.1.1. Occurrence Data

**Figure 1:**
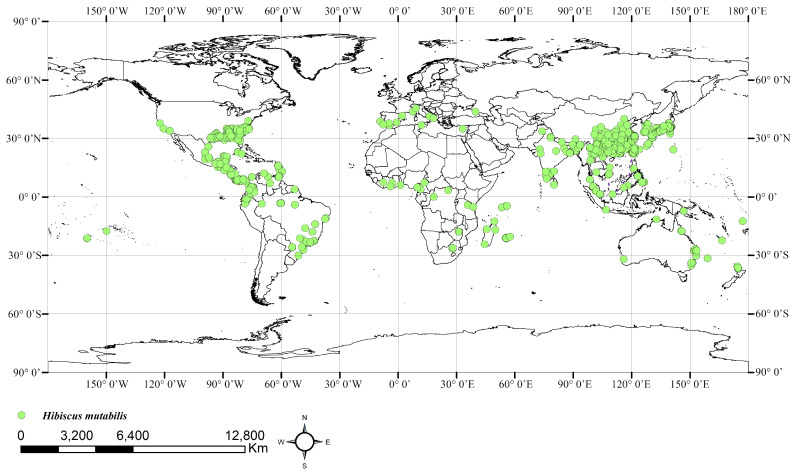
Current distribution of *H. mutabilis* in the world.

**Figure 2:**
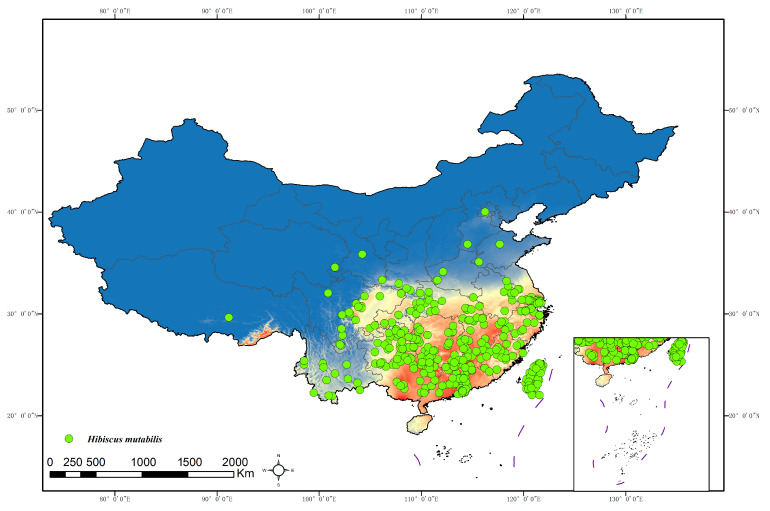
Current distribution of *H. mutabilis* in China.

The distribution data for *H. mutabilis* were primarily sourced from the Global Biodiversity Information Facility (GBIF, http://www.gbif.org, accessed on 20 May 2024), the Chinese Virtual Herbarium (CVH), and the National Specimen Information Infrastructure (NSII). The collected distribution data were processed and filtered as follows: (1) Duplicate records were removed, and for records with specific locations but without coordinate information, latitude and longitude coordinates were obtained using Google Earth. (2) To avoid spatial autocorrelation, the collected distribution data were imported into the ArcGIS system for buffer analysis. For distribution points less than 10 km apart, only one point was randomly retained. Ultimately, we obtained 985 valid distribution records of *H. mutabilis* worldwide and 374 valid distribution records in China (Figure 1 and Figure 2). Data on the distribution of *H. mutabilis* in China (Figure 2) were used for modeling according to the methodology of Du [27] and Zhang [28].

#### 2.1.2. Predictor Variables

The contemporary and future climatic data used in this study were sourced from the WorldClim database (http://www.worldclim.org, accessed on 20 May 2024). The climate data for the contemporary period (1970–2000) and three future periods (2050s, 2070s, and 2090s) were obtained using the BCC-CSM2-MR climate model. The selected climate scenarios include low forcing (SSP1-2.6), medium forcing (SSP2-4.5), and high forcing (SSP5-8.5) [29].

### 2.2. Data Processing and Selection

#### 2.2.1. Bioclimatic Variables Screening

To increase the accuracy of the simulation and prediction results, we first conducted a preliminary simulation of the distribution points of *H. mutabilis* using MaxEnt v.3.4.4 combined with 19 bioclimatic factors, then excluded bioclimatic factors with a contribution rate of 0 [30]. Subsequently, we used ArcGIS 10.8 to extract the values of the remaining bioclimatic factors for the valid distribution points of *H. mutabilis*. Pearson correlation analysis was performed on the extracted values using SPSS Statistics 22.0. When the absolute value of the correlation coefficient between two variables |r| was less than 0.8, the correlated variables were retained. When |r| exceeded 0.8, the variables were selected based on their contribution rates to the model and their permutation importance [31]. The final set of bioclimatic factors was then used to study the contemporary and future suitable habitat distribution of *H. mutabilis* in China.

#### 2.2.2. Species Distribution Model Parameter Setting

The potential distribution of *H. mutabilis* was mapped using MaxEnt version 3.4.4. In the model, 75% of the distribution points were used as training data, while the remaining 25% were used as test data, with other settings kept at their default values [32,33]. The MaxEnt model includes a jackknife test to analyze the contribution and importance of environmental variables. It uses the area under the receiver operating characteristic curve (AUC) to estimate model accuracy. The AUC value ranges from 0 to 1, with a model AUC value exceeding 0.8 considered to indicate excellent accuracy [34].

#### 2.2.3. Prediction of Potential Suitable Habitats

The asc format files from the MaxEnt model results were imported into ArcGIS software(10.8 version). Following the classification methods of Li and Deng et al. [35,36], the suitable habitats were divided into four categories: non-suitable (*p* ≤ 0.2), low suitability (0.2 < *p* ≤ 0.4), medium suitability (0.4 < *p* ≤ 0.6), and high suitability (*p* > 0.6). Using ArcGIS, the data were converted to raster format and reclassified. A map of China was used as the base map to visualize the potential distribution of *H. mutabilis* for the present, 2050s, 2070s, and 2090s. The area of potential suitable habitats for each period was calculated using the spatial analysis module in ArcGIS.

#### 2.2.4. Changes in the Area and Shifts in the Distribution Center of Suitable Habitats for *H. mutabilis*

Using the current suitable habitat area as a reference, the MaxEnt model was employed to cross-validate the suitable habitats under past and future scenarios. This approach was used to predict and calculate the changes in suitable habitat area for *H. mutabilis* across different scenarios. Subsequently, by tracking the changes in the centroid of the habitat layers, the distribution centers and migration routes of suitable habitats for *H. mutabilis* from the present to nine future scenarios were determined.

## 3. Results

### 3.1. Model Accuracy Evaluation

**Figure 3:**
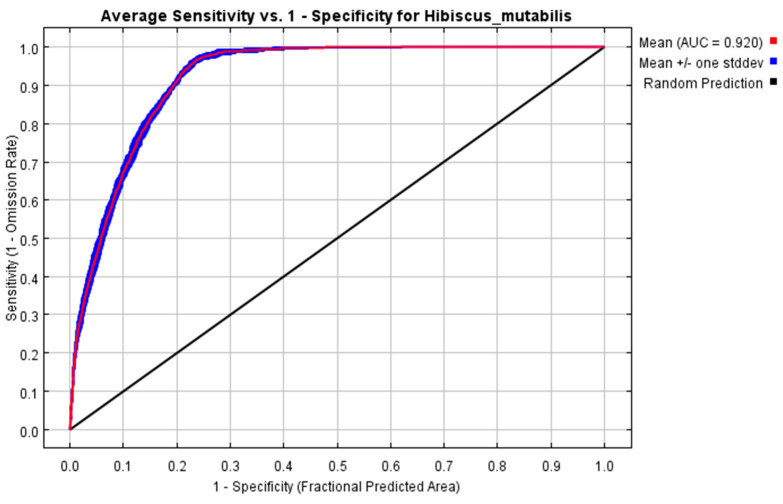
ROC prediction results of MaxEnt model.

In the current scenario, the ROC curve results indicate an AUC value of 0.920 (Figure 3), significantly higher than the random prediction value of 0.5. In the output results for the three different climate scenarios across three future periods, the AUC values for both the training and testing datasets are also above 0.9. This demonstrates that the MaxEnt model is stable and reliable, making it suitable for accurately predicting the suitable habitats for *H. mutabilis* in China.

### 3.2. Key Environmental Factors Influencing the Distribution of Suitable Habitats for H. mutabilis

We retained all 19 bioclimatic factors after the pre-simulation results showed that all of them had positive contribution rates. Subsequently, factors with absolute correlation values greater than 0.8 were removed, resulting in 11 climatic factors retained for further analysis. According to the MaxEnt model’s contemporary prediction for *H. mutabilis* (Table 1), the precipitation of the driest month (bio14) had the highest contribution rate, followed by annual precipitation (bio12), with these two factors accounting for 82.2% of the total contribution. The contribution rates of mean annual temperature (bio1), temperature annual range (bio7), mean diurnal range (bio2), precipitation of the coldest quarter (bio19), and precipitation of the warmest quarter (bio18) were relatively low, being 3.4%, 2.4%, 2.2%, 2.2%, and 2%, respectively. The lowest contribution rates were observed for precipitation of the wettest quarter (bio8), isothermality (bio3), mean temperature of the warmest quarter (bio10), and precipitation seasonality (bio15), all of which were below 2%. These results indicate that precipitation-related bioclimatic factors accounted for 89.3% of the total contribution, while temperature-related factors accounted for 10.7%. This suggests that precipitation is the key environmental variable influencing the growth of *H. mutabilis* and plays a decisive role in its geographical distribution.

**Figure 4:**
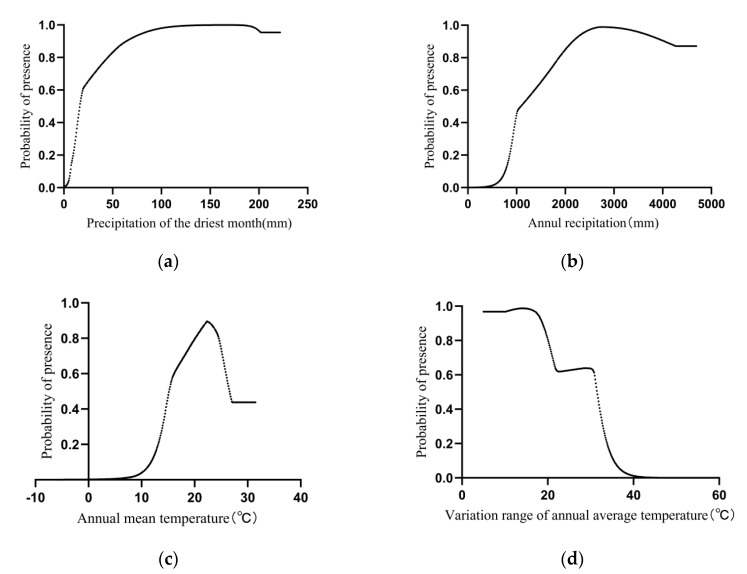
Response curves of main bioclimatic factors. (**a**) the relationship between precipitation of the driest month(X-axis) and the probability of the *H. mutabilis* distribution(Y-axis); (**b**) shows the relationship between annul precipitation(X-axis) and the probability of the *H. mutabilis* distribution(Y-axis); (**c**) shows the relationship between annul mean temperature(X-axis) and the probability of the *H. mutabilis* distribution(Y-axis); (**d**) shows the relationship between variation range of annul average temperature(X-axis) and the probability of the *H. mutabilis* distribution(Y-axis).

The response curves of the main climatic factors (Figure 4) indicate a positive correlation between the probability of *H. mutabilis* presence and precipitation. When the precipitation of the driest month exceeds 19.55 mm, the habitat becomes more suitable for *H. mutabilis* growth, with a presence probability greater than 0.6 [25]. When the precipitation of the driest month ranges from 64.16 to 222.20 mm, the presence probability exceeds 0.9, indicating optimal growth and distribution conditions. Regarding annual precipitation, when it exceeds 1372.96 mm, the presence probability is greater than 0.6, peaking at around 0.989 when annual precipitation reaches 2791.76 mm. After this point, the probability starts to decline, but remains suitable for survival. Additionally, temperature also plays a role in the growth of *H. mutabilis.* The presence probability is high when the mean annual temperature ranges from 16.22 °C to 25.85 °C, peaking at approximately 0.81 at 24.47 °C. When the annual temperature range is between 5 °C and 30.95 °C, the presence probability exceeds 0.6, peaking at around 0.987 when the annual temperature range is 14.79 °C.

### 3.3. Predicting the Suitable Habitat Range of H. mutabilis under Climate Change

#### 3.3.1. Prediction of Contemporary Potential Habitats for *H. mutabilis*

**Figure 5:**
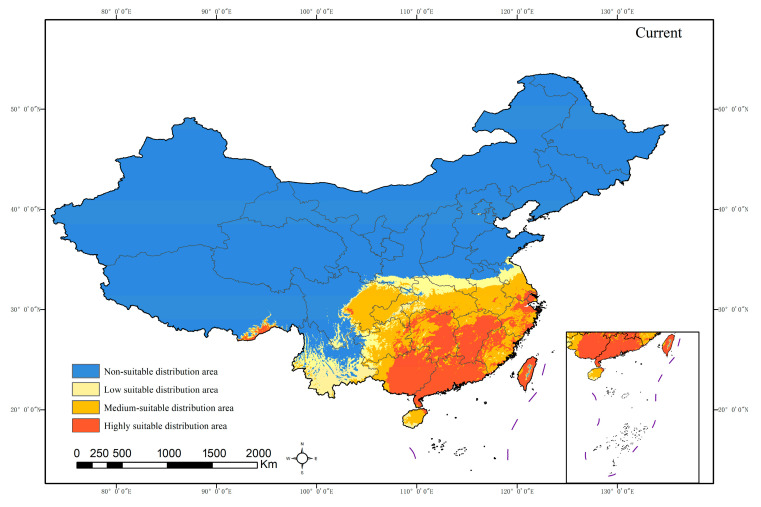
Current potential distribution area of *H. mutabilis*.

The simulation results of the MaxEnt model for the potential suitable habitat of *H. mutabilis* in contemporary China (Figure 5) indicate that the potential moderately to highly suitable habitat is mainly distributed in the southern regions, including Guangdong, Guangxi, Hainan, and Taiwan. In addition, it extends to eastern regions such as Fujian, Zhejiang, Jiangxi, and Shanghai, as well as most parts of Jiangsu and Zhejiang Provinces. Moderate to highly suitable habitats are also observed in central regions such as Hunan, Hubei, and southern parts of Henan, as well as in southwestern regions including Chongqing, eastern and southern parts of Sichuan, Chengdu Plain, and most parts of Guizhou, except the northwest, which borders Yunnan Province. The total area of potential moderately to highly suitable habitats is approximately 158.72 × 10^4^ km^2^ (Table 2).

#### 3.3.2. Future Potential Habitat Prediction for *H. mutabilis*

**Figure 6:**
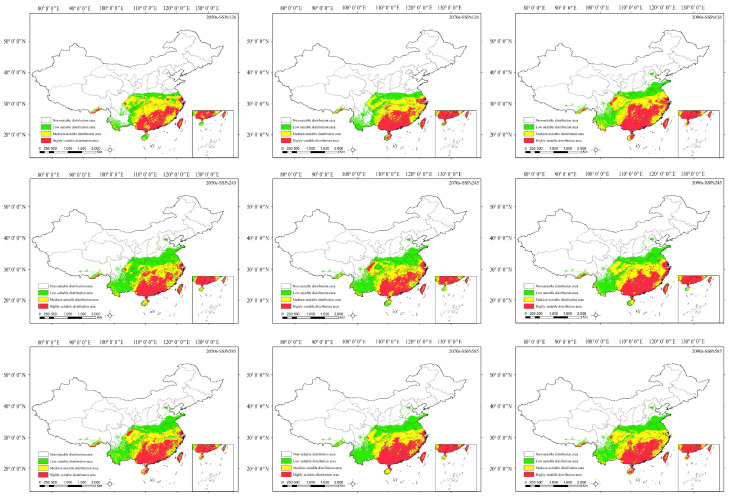
Optimum distribution of *H. mutabilis* in China under future climate scenarios at different periods.

In this study, we employed the MaxEnt model to predict the potential suitable habitats of *H. mutabilis* under three climate scenarios (SSP1-2.6, SSP2-4.5, and SSP5-8.5) for the 2050s, 2070s, and 2090s. Spatial distribution maps of potential suitable habitats (Figure 6) and the changes in habitat area for each category are presented in Table 2.

From Figure 6, it is evident that in the forthcoming decades, the moderately to highly suitable habitats of *H. mutabilis* will be primarily concentrated in provinces such as Guangxi, Guangdong, Fujian, Jiangxi, Hunan, and Zhejiang, with a tendency for the highly suitable habitats to shift southward and contract. Under the SSP126 and SSP245 climate scenarios, the area of suitable habitats will initially decrease and then increase. Specifically, by the 2050s and 2070s, the area of highly suitable habitats will decrease by 13.06 × 10^4^ km^2^ and 7.64 × 10^4^ km^2^ and 7.64 × 10^4^ km^2^ and 3.06 × 10^4^ km^2^, respectively. By the 2090s, under the SSP245 scenario, the area of highly suitable habitats will increase by 5.13 × 10^4^ km^2^ compared to contemporary levels. Conversely, under the SSP5-8.5 scenario, the area of highly suitable habitats will increase by 14.30 × 10^4^ km^2^ and 3.55 × 10^4^ km^2^ in the 2050s and 2070s, respectively, exhibiting an overall increasing trend followed by a decrease. By the 2090s, the area of highly suitable habitats will decrease below contemporary levels. Across all climate scenarios, the area of moderately suitable habitats exhibits an overall decreasing trend, while the area of low suitable habitats shows a significant increasing trend compared to contemporary levels.

### 3.4. Spatial Pattern Changes and Distribution Center Migration Trends of H. mutabilis Habitat under Climate Change

#### 3.4.1. Spatial Pattern Changes of *H. mutabilis* Habitat under Different Climate Scenarios

**Figure 7:**
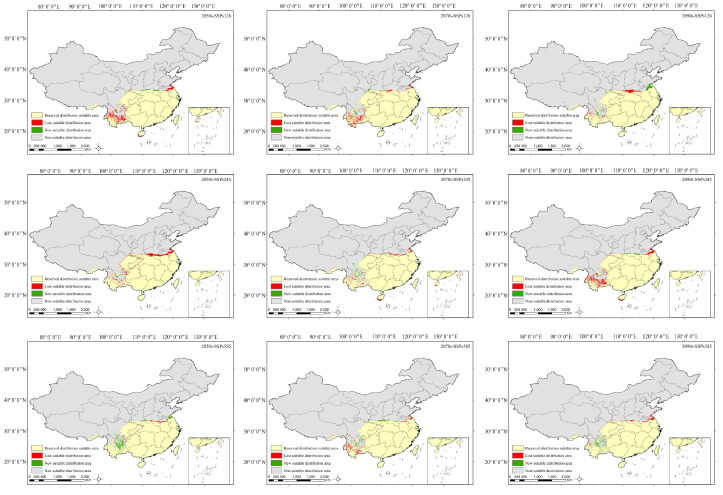
Spatial transformation pattern of the suitable area of *H. mutabilis* in different periods.

Through the spatial pattern changes of *H. mutabilis* habitat under different future climate scenarios (Figure 7, Table 3), it can be observed that the majority of contemporary suitable habitats for *H. mutabilis* remain preserved, with a retention rate ranging from 95.28% to 99.28%. These preserved habitats are mainly concentrated in regions such as Guangdong, Guangxi, Taiwan, Fujian, Zhejiang, Shanghai, Hunan, Hubei, Chongqing, Guizhou, eastern Sichuan, Chengdu Plain, southern Shaanxi and Henan, and southern Anhui and Jiangsu, among others. Under the SSP1-2.6 and SSP2-4.5 climate scenarios, the loss rates of suitable habitats exceed the increase rates during the 2050s and 2070s. The areas where habitats are lost are primarily concentrated in central Henan, northern Jiangsu, and northern Anhui. However, by the 2090s, under the SSP2-4.5 scenario, the increase rate of suitable habitats will exceed the loss rate, with new habitats mainly distributed in belt-shaped patterns across Jiangsu, Anhui, Henan, and Shaanxi, as well as in scattered patterns in Sichuan and Yunnan. Under the SSP5-8.5 climate scenario, the increase rates of suitable habitats will exceed the loss rates during the 2050s and 2090s. In particular, during the 2050s, the area of newly suitable habitats will increase by 7.46 × 10^4^ km^2^, with the highest increase rates mainly concentrated in the northern parts of Jiangsu Province, southern parts of Sichuan Province, and northern parts of Yunnan Province. However, during the 2070s, the loss rate of suitable habitats will exceed the increase rate, with scattered areas of habitat loss in Yunnan Province, northern Jiangsu Province, and northern Anhui Province.

#### 3.4.2. Trends in *H. mutabilis* Distribution Center Migration under Different Climate Scenarios

**Figure 8:**
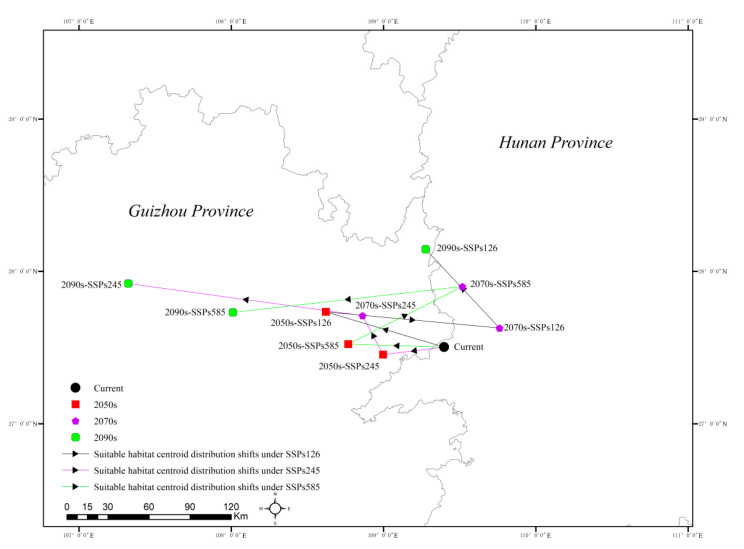
Centroid migration of *H. mutabilis* under future climate scenarios.

As depicted in Figure 8, under contemporary climatic conditions, the distribution center of *H. mutabilis* is located in Huaihua City, Hunan Province (109°23′53″ E, 27°30′08″ N).

Under the SSP1-2.6 climate scenario, in the 2050s, the distribution center will shift to Jiangkou County, Tongren City, Guizhou Province (108°37′15″ E, 27°44′01″ N), moving approximately 80.95 km northwest compared to the contemporary center. By the 2070s, the distribution center will revert to Zhijiang Dong Autonomous County, Huaihua City, Hunan Province (109°45′42″ E, 27°37′40″ N), shifting about 38.81 km northeast compared to the contemporary center. In the 2090s, the distribution center will relocate to Songtao Miao Autonomous County, Tongren City, Guizhou Province (109°45′42″ E, 27°37′40″ N), approximately 72.84 km from the contemporary center.

Under the SSP2-4.5 climate scenario, in the 2050s, the distribution center will be situated in Wanshan District, Tongren City, Guizhou Province (108°59′49″ E, 27°27′10″ N), approximately 40.45 km from the contemporary center. By the 2070s, the distribution center will move to Jiangkou County, Tongren City, Guizhou Province (108°51′44″ E, 27°42′33″ N), about 57.10 km northwest of the contemporary center. In the 2090s, the distribution center will shift to Suiyang County, Zunyi City, Guizhou Province (107°19′23″ E, 27°55′10″ N), moving approximately 210.92 km northwest from the contemporary center.

Under the SSP5-8.5 climate scenario, in the 2050s, the distribution center will be located in Jiangkou County, Tongren City, Guizhou Province (108°46′05″E , 27°31′18″ N), approximately 63.36 km west of the contemporary center. By the 2070s, the distribution center will move to Fenghuang County, Xiangxi Tujia and Miao Autonomous Prefecture, Hunan Province (109°31′03″ E, 27°54′01″ N), shifting about 84.93 km northeast compared to the contemporary center. In the 2090s, the distribution center will relocate to Sinan County, Tongren City, Guizhou Province (108°00′37″ E, 27°43′47″ N), moving approximately 150.25 km northeast from the contemporary center. Overall, under different climate scenarios, the distribution center of *H. mutabilis* exhibits a tendency towards westward migration in the 2050s, followed by a general northward trend in the 2070s and 2090s.

## 4. Discussion

### 4.1. Key Climatic Factors Restricting the Distribution of H. mutabilis

This study reveals that the precipitation of the driest month is the most crucial environmental variable influencing the distribution of *H. mutabilis*, with the other three variables being annual precipitation, mean annual temperature, and temperature annual range, which are also significant factors affecting its distribution. Within a certain range, the probability of *H. mutabilis* presence is positively correlated with the driest month’s precipitation. When the driest month’s precipitation is less than 9.373 mm, it is not suitable for the survival of *H. mutabilis*. However, when the driest month’s precipitation exceeds 64.16 mm, the probability of *H. mutabilis* presence is extremely high. Additionally, when the annual precipitation ranges from 1372.96 mm to 2791.76 mm, the probability of *H. mutabilis* presence is directly proportional to the annual precipitation. When the annual precipitation is less than 852.39 mm, it is not suitable for the survival and distribution of *H. mutabilis*. Conversely, when the annual precipitation exceeds 2791.76 mm, although the probability of *H. mutabilis* presence slightly decreases, it still remains highly suitable for its distribution. These findings indicate that the growth and distribution of *H. mutabilis* are influenced by precipitation, with drought being a limiting factor for its distribution range. Previous studies have suggested that *H. mutabilis* is intolerant to drought and tolerant to waterlogging conditions [37], exhibiting the development of adventitious roots adapting to flooded environments [24,38] without affecting flowering or ornamental characteristics. However, drought adversely affects seed germination efficiency and plant vigor [39,40], further highlighting its high water demand, which is consistent with the results of this study.

In addition to precipitation, the mean annual temperature and temperature annual range are also important climatic factors influencing the distribution of *H. mutabilis*. The MaxEnt model prediction results indicate that a mean annual temperatures exceeding 16.2 °C is highly suitable for the survival and distribution of *H. mutabilis*, with the probability of its presence peaking at 24.5 °C. Beyond this threshold, the probability of its presence noticeably declines. Conversely, mean annual temperatures ranging from −5.8 °C to 13.04 °C are not suitable for the survival and distribution of *H. mutabilis*. Studies have shown that the greatest challenge faced by *H. mutabilis* when introduced in northern regions is the inability to safely overwinter [41]. The results of this study suggest that mean annual temperature may be a key factor limiting the introduction of *H. mutabilis* to northern regions of China.

Temperature annual range also significantly influences the survival and distribution of plants. Combining response curves of climatic factors, it is observed that the probability of *H. mutabilis* presence is inversely proportional to temperature annual range. As temperature annual range increases, the probability of *H. mutabilis* presence markedly decreases. When the temperature annual range reaches 33.87 °C, the probability of *H. mutabilis* presence is less than 0.2, indicating that environments with relatively stable temperatures are more suitable for its growth.

### 4.2. Spatial Pattern Changes of H. mutabilis Habitat and Distribution Center Migration

The sixth phase of the Coupled Model Intercomparison Project (CMIP6), organized by the World Climate Research Programme (WCRP), represents the highest level of climate model simulation and prediction. With numerous participating models, scientifically designed experimental methods, and vast simulated data, CMIP6 provides more reliable estimates. Our study, based on the CMIP6 framework, effectively reveals the spatial pattern changes of *H. mutabilis* habitats and the migration of its distribution center under three new Shared Socioeconomic Pathway (SSP) scenarios. Under the SSP1-2.6 and SSP2-4.5 scenarios, the habitat area decreases in the 2050s and 2070s, but increases in the 2090s. Previous studies indicate that with the increasingly evident trend of global climate warming, future temperatures and precipitation are expected to increase compared to contemporary conditions [42]. However, the actual conditions may be more complex [43,44,45]. For instance, in the SSP2-4.5 climate scenario, Wen et al. [46] pointed out that, in the mid-to-late 21st century (2050s and 2070s) in the Yangtze River Basin, temperatures will increase while precipitation decreases, but by the late 21st century (2090s), precipitation will increase by 6% and 4.7%, respectively. According to the relationship between climate factors and the probability of *H. mutabilis* presence, it can be inferred that temperature and precipitation will synergistically affect and alter the spatial distribution pattern of *H. mutabilis*, with precipitation playing a more critical role. In future periods, extreme precipitation events in southeastern China are expected to increase, with the probability under SSP1-2.6 being lower than that under SSP5-8.5 [47]. Data from relevant research institutions indicate that under the SSP1-2.6, SSP2-4.5, and SSP5-8.5 scenarios, future temperatures will rise by 2 °C, 3 °C, and 5°C, respectively. Higher temperatures and increased precipitation resulting from high emission levels will, to some extent, favor the distribution of *H. mutabilis*. However, under future climate scenarios, extreme precipitation events are expected to be more complex, with droughts becoming more frequent. Therefore, attention should be paid to regional and sudden drought events during the introduction, domestication, and cultivation management of *H. mutabilis* [43].

Research suggests that species in the northern hemisphere may increasingly migrate to higher latitudes with the trend of global warming intensification [48]. According to the predictive results of this study, the contemporary potential distribution center of *H. mutabilis* is located in Zhijiang Dong Autonomous County, Hunan Province (109°23′53″ E, 27°30′08″ N). This region belongs to a subtropical monsoon humid climate zone characterized by mild climate, concentrated rainfall, and an annual average temperature ranging from 15.8 °C to 17.3 °C, with annual precipitation between 1156.4 mm and 1432.9 mm, making it highly suitable for the survival and distribution of *H. mutabilis*.

Furthermore, under different climate scenarios, the future distribution center of *H. mutabilis* shows a trend of westward and northward migration, mainly appearing in Tongren City and Zunyi City, Guizhou Province. Although this region is significantly affected by monsoons, currently, the summer temperatures in Guizhou are lower than those in Hunan, with more frequent dry spells during the summer monsoon season [49]. Additionally, the annual precipitation is lower, making the suitability conditions inferior to those of Hunan Province. However, forecasts indicate that temperatures in Guizhou Province are generally expected to increase compared to historical levels, with a tendency for increased precipitation in the future, providing favorable conditions for the migration and survival of *H. mutabilis* [50].

### 4.3. Conservation Strategies and Recommendations

The study on species geographical distribution responses to climate change holds significant value in providing scientific data support for species conservation [51,52]. Cindy pointed out that the shared suitable distribution areas of species in different periods often become “climate refuges” for the species due to stable climates [53]. Our study reveals that the highly suitable areas for *H. mutabilis* in both contemporary and future scenarios are located in Guangdong, Guangxi, Taiwan, Zhejiang, Shanghai, Anhui, Hunan, Hubei, Chongqing, Guizhou, eastern Sichuan, and the Chengdu Plain. Establishing conservation areas or germplasm repositories for *H. mutabilis* in these regions could effectively protect its genetic diversity. The distribution centers of *H. mutabilis* in contemporary and future climate scenarios are mainly located in Hunan and Guizhou provinces, making them key areas for *H. mutabilis* breeding. Additionally, the data indicate no records of *H. mutabilis* distribution in Hainan Province currently. However, predictive results suggest that most areas in Hainan Province will be highly suitable habitats for *H. mutabilis* under current and future climate scenarios. With an annual precipitation of 1500–2500 mm and an average annual temperature of 22.5–25.6 °C, Hainan Province possesses suitable hydrothermal conditions for the survival and distribution of *H. mutabilis*, thus warranting efforts for its introduction and cultivation.

## 5. Conclusions

Using ArcGIS software and the MaxEnt model, this study predicted the potential suitable habitats of *H. mutabilis* under nine different contemporary and future climate scenarios. The results indicate that the key limiting factor affecting the survival and distribution of *H. mutabilis* is the precipitation of the driest month, with precipitation playing a dominant role relative to temperature factors. From the perspective of potential distribution areas, *H. mutabilis* is suitable for growth and distribution in tropical and subtropical humid regions. In scenarios with low emissions (SSP1-2.6, SSP2-4.5), the area of highly suitable habitats decreases compared to contemporary levels, while in scenarios with high emissions (SSP5-8.5), the area of highly suitable habitats increases. This phenomenon may be attributed to the influence of changes in precipitation on future climate factors. The contemporary distribution center of *H. mutabilis* is located in Huaihua City, Hunan Province. Under different future climate scenarios, the distribution center mainly shifts to Tongren City and Zunyi City in Guizhou Province and Xiangxi Autonomous Prefecture in Hunan Province. In the 2050s, there will be a westward trend, while in the 2070s and 2090s, there will be a general trend of northward migration. Hainan Province has climatic conditions suitable for the growth and distribution of *H. mutabilis*, making it suitable for its introduction and cultivation. The results of this study provide guidance for the introduction, cultivation, genetic diversity conservation, and breeding management of *H. mutabilis*. However, factors other than bioclimatic factors, such as altitude and soil type, may also affect its survival and distribution. Future research will incorporate more factors into *H. mutabilis* habitat prediction to further provide a theoretical basis for the protection and scientific planting management of *H. mutabilis* germplasm resources.

## Figures and Tables

**Table 1:** Contribution of screened climatic factors to the potential distribution area of *H. mutabilis*.

No.	Bioclimatic Factor Variable	Contribution Rate/%	Permutation Importance
Bio14	Precipitation of the driest month	59.4	6.6
Bio12	Annual precipitation	22.8	13.1
Bio1	Annual mean temperature	3.4	13.1
Bio7	Variation range of annual average temperature	2.4	38.1
Bio2	Mean diurnal range	2.2	2.6
Bio19	Precipitation of the coldest quarter	2.2	0.9
Bio18	Precipitation of the warmest quarter	2	11.1
Bio8	Mean temperature of the wettest quarter	1.8	7.8
Bio3	Isothermality	1.4	2.4
Bio10	Mean temperature of the warmest quarter	1.4	2
Bio15	Coefficient of variation of precipitation	1.2	2.4

**Table 2:** Predicted area of potential habitats in different periods.

	Climate Scenarios									
Area (10^4^ km^2^)	Current	2050s			2070s			2090s		
		SSP1-2.6	SSP2-4.5	SSP5-8.5	SSP1-2.6	SSP2-4.5	SSP5-8.5	SSP1-2.6	SSP2-4.5	SSP5-8.5
LSDA	48.94	53.85	58.69	73.28	62.75	59.67	57.13	58.92	53.36	48.42
MSDA	87.02	87.07	78.93	88.27	69.76	78.49	80.27	82.57	76.06	88.61
HSDA	71.70	58.64	64.06	85.99	66.12	67.60	75.25	70.97	76.83	71.59
TSDA	207.66	199.56	201.68	247.55	198.63	205.75	212.65	212.46	206.25	208.63
HSDA-Current		−13.06	−7.64	14.30	−5.58	−4.10	3.55	−0.73	5.13	−0.11
HSDA/TSDA (%)	0.35	0.29	0.32	0.35	0.33	0.33	0.35	0.33	0.37	0.34
TSDA-Current		−8.10	−5.98	39.89	−9.03	−1.91	4.98	4.80	−1.41	0.96

Note: LSDA represents low-suitable distribution area; MSDA represents medium-suitable distribution area; HSDA represents highly suitable distribution area; TSDA represents total suitable distribution area; HSDA-Current represents the change in the highly suitable area compared to current times; TSDA-Current represents the change in the total suitable area compared to current times; SDA/TSDA (%) represents the proportion of highly suitable distribution area to total suitable distribution area.

**Table 3:** Spatial transformation pattern of the suitable area of *H. mutabilis* in different periods.

	Climate Scenarios								
Area (10^4^ km^2^)	2050s			2070s			2090s		
	SSP1-2.6	SSP2-4.5	SSP5-8.5	SSP1-2.6	SSP2-4.5	SSP5-8.5	SSP1-2.6	SSP2-4.5	SSP5-8.5
Reserved-SDA	197.58	197.85	205.14	199.85	202.49	202.54	202.54	205.92	204.09
Lost-SDA	10.10	9.84	2.57	7.84	5.16	5.13	5.16	1.77	3.60
New-SDA	1.96	0.89	7.46	1.77	3.20	3.69	4.63	6.71	4.52
NSDA	754.20	755.27	748.68	754.38	752.93	752.47	751.52	749.42	751.60
Loss rate (%)	4.86	4.74	1.24	3.77	2.49	2.47	2.48	0.85	1.73
Increasing rate (%)	0.94	0.43	3.59	0.85	1.54	1.78	2.23	3.23	2.18
Retention rate (%)	95.14	95.28	98.79	96.24	97.51	97.53	97.53	99.16	98.28

Note: Reserved-SDA represents reserved suitable distribution area; Lost-SDA represents lost suitable distribution area; New-SDA represents new suitable distribution area; NSDA represents non-suitable distribution area.

## Data Availability

Data are contained within the article.
